# A novel approach to non-segmented flow analysis. Part 2. A prototype high-performance analyser

**DOI:** 10.1155/S1463924688000082

**Published:** 1988

**Authors:** D. J. Malcolme-Lawes, C. Pasquini

**Affiliations:** Centre for Research in Analytical Chemistry and Instrumentation King's College London Strand London WC2 UK

## Abstract

A high-performance continuous flow analyser is described, based on gas pressure driven carrier and reagents controlled by computer switched solenoid valves. The principal characteristics of the analyser are discussed and examples of its performance are provided in the form of results obtained using a standard procedure for the determination of Cr(VI). The system was also tested in use with real samples using an ammonium ion analysis on potable and effluent water samples, and the results compared with those obtained using a segmented continuous flow method operated at the Laboratory of the Government Chemist.


Journal of Automatic Chemistry, Vol. 10, No. 1 (January–March 1988), pp. 25–30

### A novel approach to non-segmented flow analysis.
### Part 2. A prototype high-performance analyser

**D. J. Malcolme-Lawes and C. Pasquini**

*Centre for Research in Analytical Chemistry and Instrumentation, King's College London, Strand, London WC2*

*A high-performance continuous flow analyser is described, based on gas pressure driven carrier and reagents controlled by computer switched solenoid valves. The principal characteristics of the analyser are discussed and examples of its performance are provided in the form of results obtained using a standard procedure for the determination of Cr(VI). The system was also tested in use with real samples using an ammonium ion analysis on potable and effluent water samples, and the results compared with those obtained using a segmented continuous flow method operated at the Laboratory of the Government Chemist.*

### Introduction

In a recent paper [1] a novel approach to non-segmented continuous flow analysis was described in which a computer controlled valve switching system was used to permit the precise mixing of sample and reagents for selective reaction analysis. A much improved version of the apparatus has now been constructed, which permits a variety of analytes to be determined sequentially and provides excellent sample throughput and sensitivity. In this paper the elements of the instrument and its characteristics are described, and some examples of analytical results obtained using standard analytical procedures for Cr(VI) and $NH_4^+$ are presented.

### The analyser

The instrument [1] used a carrier flow of a low-cost, inert (in the sense that it does not contribute to the reaction) liquid, into which both sample and reagents were injected through computer controlled valves. Gas pressure propulsion was used for both carrier and reagents as this had the advantage of producing a liquid flow which is pulse free, giving rise to less noise in the detector signal than could be obtained from peristaltic pumps. Furthermore, gas pressure propulsion is inexpensive and eliminates the use of peristaltic tubing, which, in turn, allows virtually any reagents to be used without difficulty. The main disadvantage normally found in using gas propulsion is the lack of flow rate control when used in confluence systems. However, this is not a limitation with the present system as only one reagent valve is open at any one time – reagent mixtures are generated by rapidly switching valves. The carrier liquids and the reagents were stored separately in Schott Duran bottles (generally 1000 and 500 ml capacities respectively) fitted with similar closures to those described earlier [1]. Two PTFE tubes were connected to each reservoir using Cheminert couplings. One tube carried the pressurizing gas and terminated at a hole in the closure. The other carried the liquid from the reservoir and passed through the closure, the tube-closure seal being completed by a small silicone O-ring.

Carrier, reagent and sample flows were controlled using solenoid valves connected together as a manifold using 1/16th in od, 0·023 in id PTFE tubing. The valve connection manifold is shown in figure 1. Valves 1 and 2 allowed a choice of principal carrier liquids, while valves 8–15 allow selected reagents, diluents or alternative carriers to enter the flow by replacing the principal carrier. By operating groups of valves alternately at a frequency of 20 Hz it was possible to produce reagent mixtures in the manifold and this technique is particularly useful for reagent mixtures which would exhibit a short shelf life. A number of chemistries used for established colorimetric determinations utilize reagents which have limited lifetimes, including those for the determinations of $NH_4^+$ and Cr(VI) discussed below. The reagent or mixture selected for a particular determination was allowed to fill the manifold for a predetermined time period, referred to as the fill time ($t_f$), controlled by the computer and defined by a software procedure written for the determination. At this point the sample loop circuit (bounded by valves 4 and 5) could be switched out of the manifold and filled with sample using the solenoid operated syringe pump. Excess liquid was expelled from the syringe pump through valve 3, so the pump could be operated repeatedly to fill any desired loop volume.

Once the sample loop had been loaded the pathway between valve 15 and the reaction tube contained a reagent-sample-carrier or a reagent-carrier-sample pattern which depended on the magnitude of $t_f$ and was of precisely reproducible volumes. The first pattern gave maximum sensitivity, while the second could be used for an automatic in-line dilution type of procedure, in which only the highly dispersed portion of the sample zone mixed with reagent. For $t_f = 5{\cdot}0$ s the total reagent volume is approximately 400 μl (generally enough for the maximum sensitivity mode of operation) and relative standard deviations of 0·27, 0·30 and 0·50% were observed for eight measurements of the mass of water delivered to a weighing bottle from 1, 2 and 3 reagent bottles respectively.

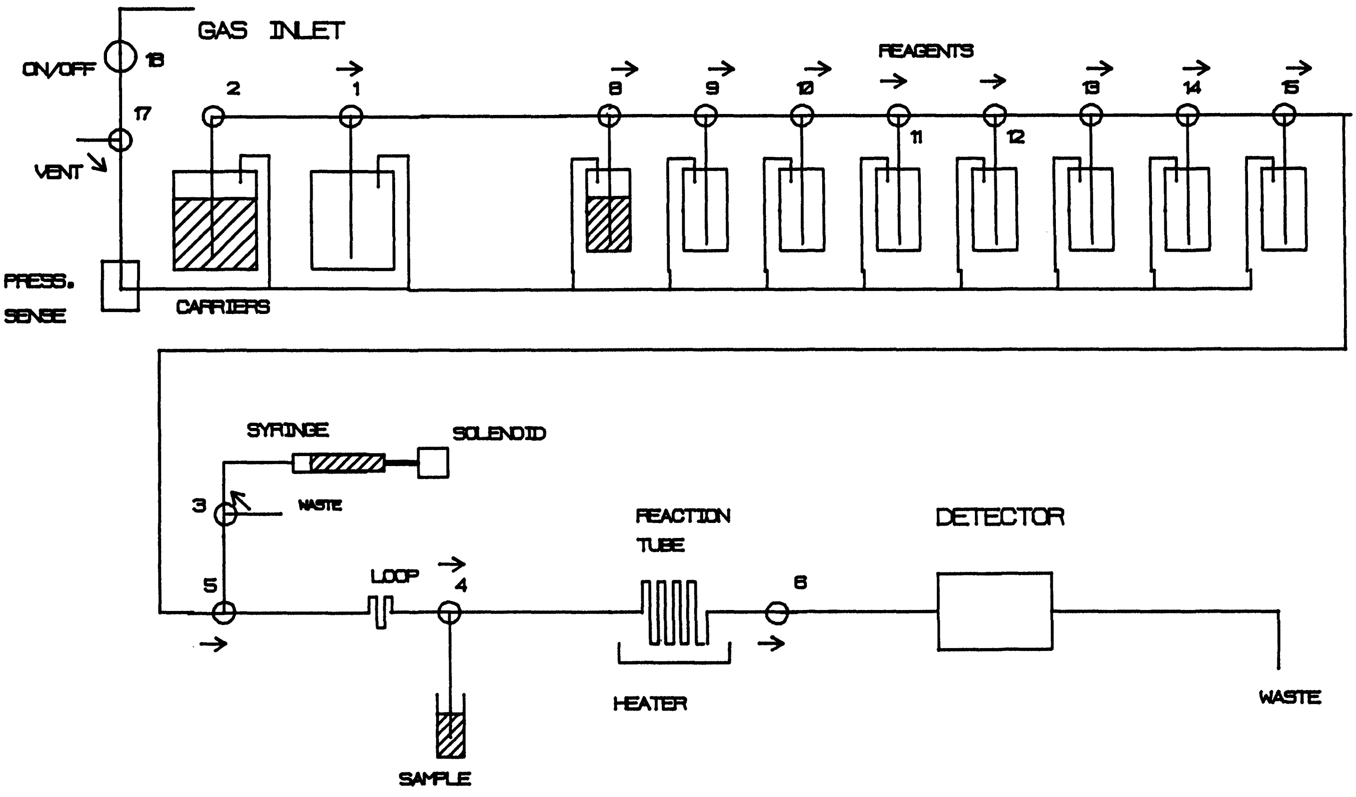

*Figure 1. Schematic diagram showing valve interconnections of the prototype instrument. The arrows show the normally open pathway through the valves.*

In the present system the sample loop volume was approximately 160 μl, and the system used up to about 400 μl of reagent mixture (when operating in maximum sensitivity mode) and a total of 400 μl of sample (including the volume in the sample delivery tube) for each analysis. This latter volume could have been made smaller, although it compares favourably with the actual sample usage in other automatic analysis systems. Closure of valves 4 and 5 caused this sample-containing sandwich to be passed through to the reaction tube and ultimately to the detector. Once the reaction mixture was in the reaction tube valve 6 could be closed to stop the flow for a precisely controlled reaction time. As the reaction tube was maintained at a user-specified temperature, a wide variety of reaction times and temperatures could be employed to accommodate a range of reaction chemistries. Certainly if the reaction was carried out at an elevated temperature, it was desirable that valve 6 should be closed to avoid the formation of gas bubbles within the reaction tube. After a timed reaction period valve 6 was opened and the reaction mixture passed through the detector system and to waste.

The only important volumes, and hence lengths of interconnecting tubing, were for the segment between valves 4 and 6 (the reaction tube), and that between valves 4 and 5 (the sample loop). In the present system these volumes were 160 and 750 μl respectively, obtained by using lengths of 25 cm of 0·035 inch id, and 150 cm of 0·032 inch id tubing. Other interconnecting tubes were maintained as short as is practical, allowing for considerable economy in the use of reagents.

The instrument was fitted with a purpose-made, variable wavelength, single beam absorbance monitor which had its monitoring wavelength set by the controlling microcomputer. The unit incorporated a flow cell with a light path length of 1 cm and an internal volume of approximately 30 μl. The system could be used to monitor the absorbance at a fixed wavelength (between 350 and 750 nm in the prototype) of a reaction mixture passing through the flow cell, or alternatively the flow could be stopped with the reaction mixture within the flow cell and the absorbance spectrum or the kinetic variation of the absorbance recorded.

The instrument's control system was based on a multifunction interface unit [2] connected to the handshaking parallel port of a microcomputer, and a Commodore 64, a BBC Master 128, and a PC clone have all been used in this role. For the PC clone a simple parallel port adaptor [3] was required. The functions of the interface circuitry are summarized in figure 2. The interface was addressable, so any of the interface functions may be operated independently, and much of the control software was written in assembler language so that speed of operation more than adequate. The interface circuitry handled the switching of the 18 valves which controlled the liquid flow and gas pressure, the solenoid which operated the sample loading syringe pump, the heater which maintained the reaction tube at the desired temperature (between room temperature and 60 °C), and the wavelength setting of the monochromator of the detector. The circuitry also controlled an indicator light on the front panel of the instrument, and this was turned on to indicate that the

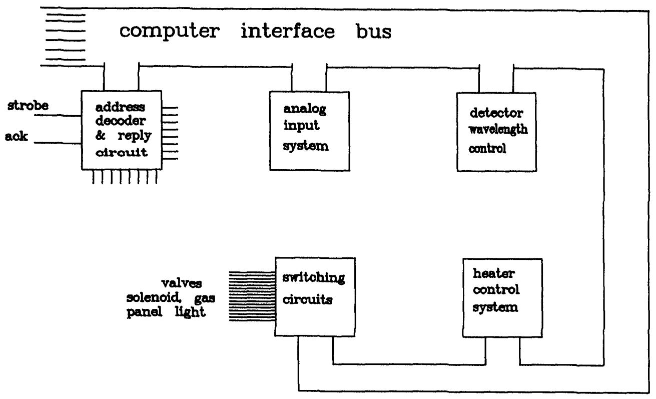

*Figure 2. Principal elements of the computer interface. The address decoder, valve switching logic buffers, heater control and analogue input circuits are incorporated in a single circuit board within the analyser.*

user could press a push-button to initiate a manual sample load – the push-button generated a dummy handshake within the interface, but only when the indicator light was on. The system also included a multiplexed dynamic ADC interface [4] which allowed a reading over the range 0–1 V with a precision of approximately 10 microvolts, and could be used to read the detector's output, the reaction tube temperature, the ambient temperature, and the system operating pressure.

The system's software allowed the user to define a procedure for specific analytical determinations. Each procedure consisted of a definition of the carrier bottle to be employed (see figure 1), the reagent bottle or bottles from which the reagents were obtained and the associated fill time, the reaction tube temperature, the wait time (i.e. the time the reaction mixture was held stationary in the reaction tube), and the detector's monitoring wavelength. The parameters used to convert from the detector's output signal to analyte concentration were determined during the calibration procedure and stored along with the procedure on disk. Two options are provided: linear least squares and interpolation, the latter being useful for those analyses which do not show linear calibration curves (such as the ammonium ion determination discussed below).

In normal operation the user loaded the required procedure from disk and was then given the choice of calibrating the procedure or carrying out analyses. Any number of replicate determinations could be specified, and procedures defined for the determination of up to eight analytes sequentially from a given sample solution. The manual presentation of samples required the user to place the instrument's sampling tube into each sample bottle when prompted and press the front panel button when ready, although it is more likely that an instrument of this kind would be operated with an autosampler or by repeatedly sampling material from a single source (for example a process flow or effluent).

During analysis the detector's output was illustrated on the computer display screen both graphically and digitally, and procedures allowed for graphics hard copy to be obtained on a chart recorder. The derived analyte concentration was normally printed on a printer along with statistical data when replicate determinations had been specified.

When the instrument was first used after power up or a bottle refill the connecting tubes were briefly flushed to expel air bubbles, and when the instrument was closed down the gas pressure within reagent bottles was released. Reagent bottles could contain sufficient reagent for 1000 determinations, so the instrument could operate for long periods without requiring manual intervention.

### Instrument performance

The instrument's dispersion coefficient [5] was determined by the comparison of the absorbances of coloured solutions (acidic copper sulphate solutions) pumped directly through the detector with the peak absorbances of loaded samples of the same solutions propelled from the sample loop by water carrier. The dispersion coefficient was measured as 2·5. This is a reasonable value considering the relationship between the reagent and sample mixing and sensitivity. Lower dispersion coefficient values may be obtained by reducing the length of the reaction coil, although this results in poor mixing, particularly when there is a large difference in density and/or viscosity between sample and reagent. Furthermore, short reaction coil lengths are not suitable for analyses in which multiple reagents are used. Higher dispersion coefficient values lower the sample throughput rate (by increasing the time for the detector signal to return to its baseline value) and lower the sensitivity (by decreasing the peak height). With the reaction coil length described, the noise level observed at the detector (operating at 790 nm) when simulated reagent mixtures of (*a*) 2% $CuSO_4$ and water; (*b*) 2% $CuSO_4$ in 1 M $H_2SO_4$ and water; and (*c*) 5 M NaOH and water, were created was in all cases approximately 0·0002 AU.

An evaluation of the precision of the instrument in the absence of chemical reaction was performed by loading $CuSO_4$ samples into water carrier and monitoring the peak height at 790 nm. The results, expressed as averages over 10 measurements together with standard deviations, were: (0·0748 ± 0·0009) AU for 0·5% $CuSO_4$, (0·1439 ± 0·0006) AU for 1·0% $CuSO_4$ and (0·2809 ± 0·002) AU for 2% $CuSO_4$ solution. These results clearly demonstrate that the sampling procedure, timing control, gas propulsion and detection systems are capable of good precision.

As an example of the instrument's performance in using a single analyte procedure some results are presented for the determination of chromium VI based on the reaction with diphenylcarbazide (dpc) under acid conditions [6]. Acidified diphenylcarbazide is relatively unstable, so two solutions from reagent reservoirs were mixed to produce the reagent prior to sample loading. One reagent was 0·06% dpc in 5% (v/v) acetone, and the other 0·8 M sulphuric acid solution in water. The carrier used for the reactions was 0·4 M sulphuric acid made 2·5% (v/v) with acetone. Water carrier could be used at other times and for washing the system. Samples were made by dilution

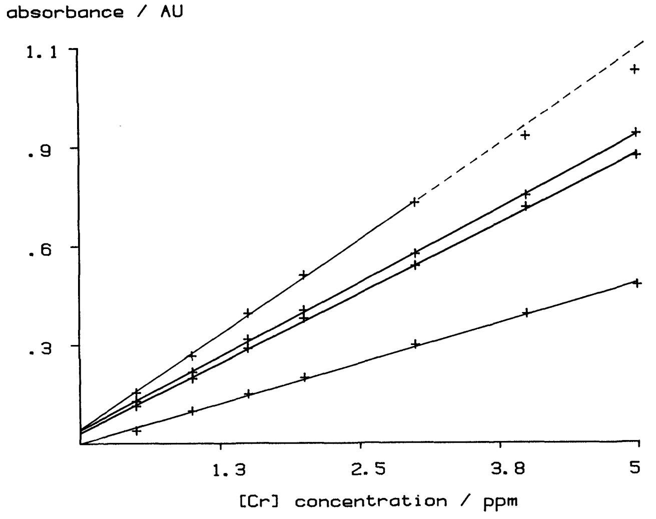

*Figure 3. Absorbance values at 545 nm recorded for Cr(VI) analyses over the concentration range 0·5–5·0 ppm, after reagent fill times of 1·0, 2·5, 3·5 and 5·5 s.*

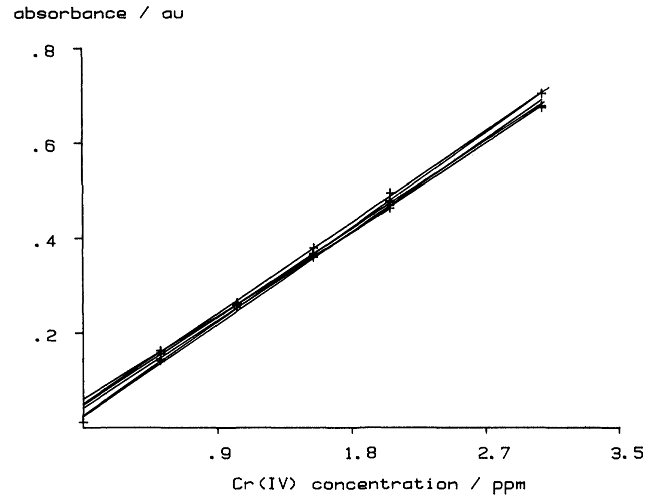

*Figure 4. Calibration of the Cr(VI) analysis over the concentration range 0·5–3·0 ppm on several different days, using a fixed reagent fill time of 10 s.*

from a stock solution of 0·1 M sulphuric acid containing 100·0 ppm Cr(VI), initially in the form of chromic oxide. The gas pressure was approximately 20 psig, producing a carrier flow rate of 5·2 $cm^3$ $min^{-1}$. The reactions were performed with the reaction tube heater maintained at 35·0 +/− 0·5 C and the absorbance was monitored at 545 nm.

Figure 3 shows a number of calibrations of the Cr(VI) analysis over the range 0·5–5·0 ppm Cr(VI) for fill times of 1·0, 2·5, 3·5, 4·5 and 5·5 s. The correlation coefficients were 0·9992, 0·9997, 0·9999, 0·9996 and 0·9991 respectively. The results show how the software controlled parameters can be used for automatic in-line dilution procedures to extend the range of maximum concentration which can be directly measured by the analyser. Figure 4 shows a number of calibration curves for the range 0·5–3·0 ppm Cr(VI) recorded on different days

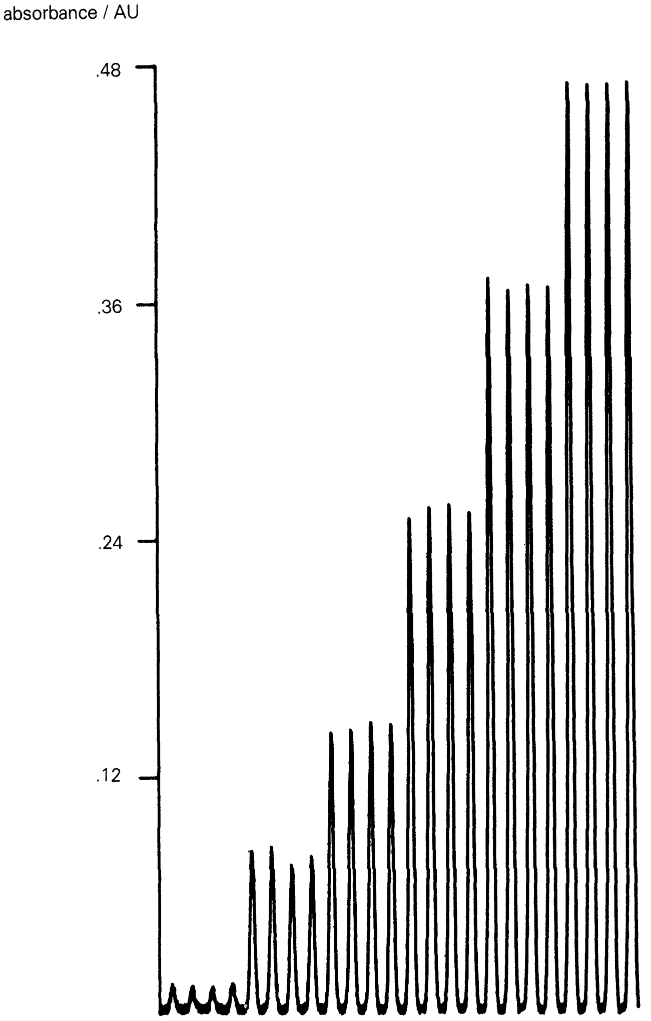

*Figure 5. Absorbance peaks (at 545 nm) derived from successive samples of a blank and 0·2, 0·5, 1·0, 1·5 and 2·0 ppm Cr(VI) solutions.*

using a fixed $t_f$ of 5·5 s to demonstrate the medium term reproducibility of the system operating in maximum sensitivity mode, while the short-term reproducibility is demonstrated in figure 5, where the peak heights recorded from successive samples of a blank and 0·2, 0·5, 1·0, 1·5 and 2·0 ppm Cr(VI) solutions are shown, and table 1, were the recorded peak heights obtained from repeated sampling of a blank and solutions of 0·2, 0·5, 1·0, 2·0 and 3·0 ppm standards. The detection limit estimated from twice the standard deviation of the blank measurement divided by the sensitivity is 0·010 ppm Cr(VI), and linear calibration was achieved up to 3 ppm in maximum sensitivity mode. The reagent consumption per Cr(VI) determination in this mode is approximately 15 fold lower than that previously reported using a conventional FIA system.

### Ammonium ion determination

Ammonium ion determinations were carried out using the Berthelot reaction catalysed by sodium nitroprusside. As was the case for the Cr(VI) determination, the

*Table 1. Peak heights recorded from Cr(VI) standards by repeated sampling.*

| Sample | 0·20 ppm | 0·50 ppm | 1·0 ppm | 1·5 ppm | 2·0 ppm |
|---|---|---|---|---|---|
| | Peak height (AU) | | | | |
| 1 | 0·0852 | 0·1580 | 0·2651 | 0·4970 | 0·6847 |
| 2 | 0·0799 | 0·1695 | 0·2666 | 0·4709 | 0·6910 |
| 3 | 0·0843 | 0·1544 | 0·2739 | 0·4936 | 0·6855 |
| 4 | 0·0846 | 0·1632 | 0·2739 | 0·4884 | 0·6803 |
| 5 | 0·0796 | 0·1557 | 0·2739 | 0·4866 | 0·6885 |
| 6 | 0·0814 | 0·1603 | 0·2763 | 0·4732 | 0·6859 |
| 7 | 0·0827 | 0·1579 | 0·2709 | 0·4794 | 0·6809 |
| 8 | 0·0865 | 0·1600 | 0·2623 | 0·4870 | 0·6846 |
| 9 | 0·0904 | 0·1581 | 0·2671 | 0·4858 | 0·6884 |
| 10 | 0·0815 | 0·1537 | 0·2690 | 0·4905 | 0·6834 |
| Mean | 0·0836 | 0·1591 | 0·2699 | 0·4852 | 0·6853 |
| Standard deviation | 0·0033 | 0·0046 | 0·0046 | 0·0084 | 0·0034 |

*Table 2. Reproducibility of the $NH_4^+$ method.*

| Sample | 0·06 ppm | 0·10 ppm | 0·20 ppm | 0·70 ppm | 1·00 ppm |
|---|---|---|---|---|---|
| | Peak height (AU) | | | | |
| 1 | 0·0367 | 0·0513 | 0·0759 | 0·2158 | 0·2982 |
| 2 | 0·0372 | 0·0523 | 0·0761 | 0·2163 | 0·2972 |
| 3 | 0·0350 | 0·0540 | 0·0769 | 0·2147 | 0·2986 |
| 4 | 0·0360 | 0·0560 | 0·0745 | 0·2139 | 0·2951 |
| 5 | 0·0360 | 0·0515 | 0·0765 | 0·2156 | 0·3015 |
| 6 | 0·0373 | 0·0525 | 0·0776 | 0·2176 | 0·2977 |
| 7 | 0·0370 | 0·0531 | 0·0741 | 0·2199 | 0·2955 |
| 8 | 0·0381 | 0·0522 | 0·0761 | 0·2172 | 0·2985 |
| 9 | 0·0350 | 0·0543 | 0·0787 | 0·2176 | 0·3002 |
| 10 | 0·0362 | 0·0529 | 0·0770 | 0·2144 | 0·2960 |
| Mean | 0·0369 | 0·0530 | 0·0763 | 0·2163 | 0·2978 |
| Standard deviation | 0·0010 | 0·0014 | 0·0014 | 0·0018 | 0·0020 |

reagents had to be stored separately to ensure reproducibility. Three solutions were employed: 0·5% phenol (w/v) containing 0·5% (w/v) sodium nitroprusside, 0·15% sodium hypochlorite solution, and a masking solution of 0·35 M NaOH containing 1·5% (w/v) EDTA (disodium salt). The carrier was 0·05 M NaOH solution. Standard solutions were prepared from a stock solution containing 100·0 ppm $NH_4^+$ as $(NH_4)_2SO_4$. The system pressure and flow rates were as described for the Cr(VI) determinations, but the reaction coil heater was operated at (50·0 ± 0·5)°C. It was determined empirically that a reaction mixture heating time of 12 s allowed complete colour development. The mixture's absorbance was monitored at 690 nm.

Table 2 shows the results of a series of repetitive measurement for the $NH_4^+$ standard solutions in the range 0·06–1·00 ppm. In this case the precision is somewhat better than that recorded for the Cr(VI) determinations, probably because the final concentration of reagents in the reaction mixture is relatively low, giving a density and refractive index similar to those of the sample. The calibration curves for the procedure do show a non-linear relationship over the range 0–0·3 ppm $NH_4^+$, with the sensitivity decreasing to high concentrations. From 0·3–1·5 ppm the calibration shows good linearity with a typical corelation coefficient of 0·9995 and a sensitivity of 0·2895 AU/ppm. Low $NH_4^+$ concentration samples were determined using the interpolation option of the computer software. The detection limit was 0·007 ppm, estimated as described for the Cr(VI) procedure.

Table 3 shows a comparison between the results obtained for potable and effluent water samples using the method described above, and those obtained using the segmented continuous flow analysis (SCFA) method operated by the Laboratory of the Government Chemist. Good agreement may be observed. Sample number 6 was determined six times using different calibration curves, and the average and standard deviation for the $NH_4^+$ concentration was (0·105 ± 0·005) ppm.

The sensitivity achieved with our present instrument is lower than that obtainable using the SCFA method – precisely because our samples are diluted 2·5 fold by dispersion. However, the precision of our results allows for a adequate detection limit and the analytical rate (80 samples per hour) is about four times greater than available using the SCFA method. When compared with previous FIA procedures [7] our results show similar sensitivities (7 ppb compared with 5 ppb in reference [7])

*Table 3. Comparison between the present work and the SCFA method for real ammonium samples. (Units: ppm.)*

| Sample | Present work | SCFA method |
|---|---|---|
| 1* | 1·21 | 1·19 |
| 2 | 0·094 | 0·096 |
| 3 | 0·064 | 0·05 |
| 4 | 0·020 | 0·03 |
| 5* | 1·08 | 1·04 |
| 6 | 0·105 | 0·105 |
| 7 | 0·061 | 0·07 |
| 8 | 0·063 | 0·04 |

Note * samples analysed after 10-fold dilution.

but show two advantages. Firstly the reagent consumption is decreased 16 fold in the case of the phenol reagent, eight fold for the nitroprusside and five fold for the hypochlorite. Secondly the sample volume used by our system is about six fold lower than that employed previously, while sample throughput has remained comparable (80/h compared with 90/h in reference [7]).

### Discussion

The results indicate that the instrument is capable of a precision, sensitivity and reproducibility at least as good as those obtained previously [6 and 7] and those which could be obtained from available commercial continuous flow analysers. However, there are a number of attractive features of the new design. For example relatively small volumes of both sample and reagents are required for each analysis, approximately 400 microlitres of reagent and 160 microlitres of sample in the present configuration, so that the present bottles of reagent can be used for approximately 1000 samples without refilling. Furthermore, alternative analyses may be carried out by employing the remaining six reagent bottles without any manual modifications to the instrument. Changing the monitoring wavelength under computer control between analyses would add only a few seconds to the analysis time.

Although the simple sampling system has only been used here in a manual mode (an automatic sample changer is currently being installed), the reproducibility of the technique is high and the wastage of sample is small. Contamination of the sample delivery tube does not seem to be a problem, although it would be a simple matter for this tube to be washed between samples (by opening valve 4) if this should prove necessary. The results reported above were all obtained using a standard cylinder head gas regulator, and we have experienced no difficulty with flow rate fluctuations. Sample throughput for the chromium analysis was approximately 100 samples/h, although this may be increased if desired by operating at higher flow rates. (Using manual sample handling it becomes difficult to cope with the physical manipulations of a faster throughput.)

Commercial versions of the instrument described are available from Biotech Instruments Ltd, 183 Camford Way, Luton LU3 3AN, UK.

### Acknowledgements

Some aspects of this work were performed with the aid of support from the Science and Engineering Research Council and of Biotech Instruments Ltd. The authors are also grateful for the advice of Dr R. Newton of Biotech Instruments and for the co-operation of Dr R. Moxon of the Laboratory of the Government Chemist in providing ammonium samples and analyses. Patent protection has been applied for to cover some aspects of the instrument described in this paper. C. P. was on leave from Universidade Estadual de Campinas – SP, Brazil. D. J. M-L is a Royal Society Research Fellow in the Physical Sciences.

### References

1. Malcolme-Lawes, D. J., Milligan, G. A. and Newton, R., *Journal of Automatic Chemistry*, **9**, 4 (1987), 179.
2. Malcolme-Lawes, D. J., *Laboratory Microcomputer*, **6** (1986), 16.
3. Malcolme-Lawes, D. J., *Laboratory Microcomputer*, **6** (1987), 82.
4. Malcolme-Lawes, D. J., *Laboratory Microcomputer* (in press).
5. Valcarcel, M. and Luque de Castro, M. D., *Flow Injection Analysis: Principles and Applications* (Ellis Horwood Ltd, Chichester), (1987).
6. de Andrade, J. C., Rocha, J. C., Pasquini, C. and Baccan, N., *Analyst*, **108** (1983), 621.
7. Krug, F. J., Reis, B. F., Gine, M. F., Zagatto, E. A. G., Ferreira, J. R. and Jacintho, A. O., *Analytica Chimica Acta*, **151** (1983), 39.

**SEVENTH INTERNATIONAL SYMPOSIUM ON MASS SPECTROMETRY IN LIFE SCIENCES**

*To be held in Ghent, Belgium from 23 to 26 August 1988*

The symposium is being sponsored by the Faculty of Pharmaceutical Sciences of the State University of Ghent, the National Foundation of Scientific Research (N.F.W.O.-F.N.R.S.) and the Ministry of National Education of Belgium. Contributed papers and posters will cover the following topics: Fundamental aspects and applications of mass spectrometry in biological, environmental and health sciences; New developments in instrumentation and techniques of analysis; Qualitative and quantitative applications in drug analysis, pharmacokinetics, clinical chemistry, biochemistry, toxicology, environmental and ecological research. All papers must be presented in English and no simultaneous translation will be provided. The deadline for receipt of abstracts is 15 May 1988.

*Further information from Professor Dr A. De Leenheer, Laboratoria voor Medische Biochemie en voor Klinische Analyse, Harelbekestraat 72, B 9000 Gent, Belgium.*

